# Study on Ultrasonic Imaging of Nursing Care for Preventing and Treating Clinical Infection of Hemodialysis Patients Based on Smart Medical Big Data

**DOI:** 10.1155/2021/2551063

**Published:** 2021-12-06

**Authors:** Yuanyuan Wen, Hongyan Li, Yanjun Gao

**Affiliations:** ^1^Nephrology Blood Purification Center, Beijing Jishuitan Hospital, Beijing 100096, China; ^2^Nursing Department, PLA Naval General Hospital, Beijing 100048, China

## Abstract

The ultrasonic imaging research of nursing care for preventing and treating clinical infection of hemodialysis patients based on smart medical big data is studied. 100 hemodialysis patients were selected from May 2019 to May 2020. The patients were randomly divided into the observation group and routine group with 50 cases in each group. The PWV of common carotid artery was measured by ultrasonic rapid imaging technology, including BS value at the beginning of systole and ES value at the end of systole. According to the effect of preventive nursing intervention of intelligent medical treatment, the MHD group adopted preventive nursing intervention, while the routine group adopted traditional nursing service. The infection rate and quality of life score of patients in both groups were evaluated. The results showed that there were significant differences in BS and ES values between the MHD group and PWV in the normal group (*P* < 0.05). There was no significant difference in BS value and ES value between MHD patients with plaque and those without plaque (*P* > 0.05). It is proved that the ultrafast ultrasound imaging technology is safe, simple, noninvasive, nonradioactive, and fast and can automatically and accurately detect carotid PWV. It is expected to become a new imaging method for quantitative evaluation of arteriosclerosis degree in MHD patients. Preventive nursing intervention can reduce the incidence of infection in hemodialysis patients and improve their quality of life. Smart medical treatment has brought us a lot of convenience. As patients, we should change our concept, actively participate in it, and contribute to the development of smart medical treatment.

## 1. Introduction

With the continuous progress of society, people's living standards have improved significantly, and the medical and health problems are closely related to people's quality of life. However, there are still many problems in the medical and health fields, such as difficult and expensive medical treatment. With the rapid development of information technology, medical informationization has shown an unprecedented development trend. The close combination of technology and medical treatment has promoted the reform and innovation of medical industry. In recent years, the emergence of new information technologies based on Internet of Things, big data, cloud computing, and mobile Internet has gradually penetrated into the development and life of cities, thus providing favorable solutions for medical and health problems [1]. Maintenance hemodialysis refers to the use of hemodialysis to save the lives of patients, which is an important method to prolong the lives of patients with end-stage kidney disease [2]. In recent years, with the continuous development of hemodialysis technology, there are fewer and fewer complications in the treatment of end-stage kidney disease, and cardiovascular disease is the most common cause of death for 50% of MHD patients. Cardiovascular events are always accompanied by changes in vascular structure and function, and arterial elasticity is one of the indexes reflecting the state of vascular function. Therefore, early prediction of arterial elasticity changes in MHD patients is of great significance. The main methods for evaluating arterial elasticity include measuring pulse pressure, two-dimensional strain, pulse wave velocity and echo-tracking, and PWV is considered as an important index, which can accurately reflect arterial elasticity [3].

The economic development has been rapidly improved, and the people's living standards have been continuously improved. Medical problems are closely related to people's health [4]. However, there are always great problems in the field of medical and healthcare, such as “it is difficult and expensive to see a doctor,” which is the biggest problem in medical and healthcare. For the shortage and unreasonable allocation of medical and health resources, rich medical resources are concentrated in big hospitals in big cities, while grassroots health service institutions are very weak. The medical quality is low, and the safety of patients has problems. The nature of public hospitals has changed to profitability, and the expensive medical expenses are difficult for patients to bear. People's growing demand for medical care contradicts the quality of medical services. Therefore, a wide range of reforms are needed in the field of medical and healthcare, which is in order to be truly patient-centered [5]. With the increasing attention in the field of medical and healthcare, the new term “smart medical care” was born. The platform architecture of digital intelligent medical ward is designed by Marsenic O. The diagnosis and treatment service mode of digital ward is innovated, and the quality control system of digital intelligent medical ward is constructed [6]. Li J. studied the design of regional medical information sharing platform and provided technical support for regional medical information construction [7]. Cao J. mainly studied the application status, development prospect, and value realization of health big data and put forward the basic framework of health big data analysis [8]. In this study, the PWV at the beginning of systole and at the end of systole was directly measured by tracing the movement of the anterior wall of carotid artery, and the clinical application value of detecting the hardness of common carotid artery in hemodialysis patients was discussed.

## 2. Materials and Methods

### 2.1. The Basic Concepts of Smart Medical Care

**Figure 1:**
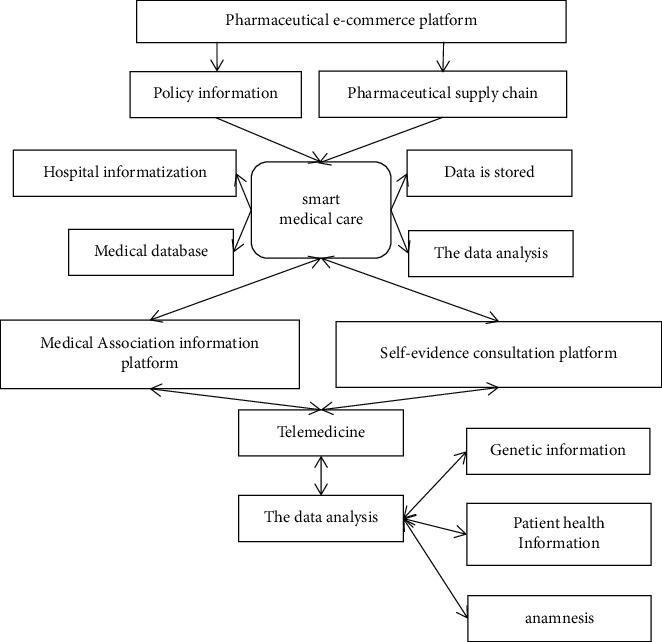
Concept map of smart medical treatment.

With the continuous progress of science and technology and the rapid development of information technology, the medical and health field is gradually moving towards informationization and intelligence, and the concept of “smart medical care” comes into being with the progress of society. WIT120 (wisdom medical treatment) is a new proper medical term. At present, the concept of smart medical care in the industry is still in the exploratory stage, and each concept has its own emphasis. Medical informatization presents an unprecedented development trend, and the close combination of technology and medical care promotes the reform and innovation of medical industry. In recent years, the emergence of new information technologies based on the Internet of Things, big data, cloud computing, and mobile Internet has gradually penetrated into the development of urban life. Somebody believes that smart medical care refers to the use of Internet of Things technology to realize the interaction between patients and medical personnel, medical institutions, and medical equipment, which will promote the innovation of medical information mode, and finally realize real-time, intelligent, automated, and interconnected dynamic services. Smart medical care refers to a medical system that integrates medical infrastructure and IT infrastructure through medical Internet of Things, medical cloud, mobile Internet, data fusion, data mining, and wearable devices and makes intelligent decisions on this basis, thus overcoming the time and space constraints and technical constraints of the original medical system and realizing the optimization of medical services [9]. According to IOT information, the smart medical treatment is to realize the interaction between patients, medical personnel, medical institutions, and medical equipment by building a regional medical information platform for health records and using the most advanced Internet of Things technology and gradually achieve informationization. We believe that smart medical care refers to the use of Internet of Things, cloud computing, data mining, and other related information technologies, which will closely link patients with medical personnel, medical equipment, and medical institutions, promote the comprehensive informationization mode of medical treatment, and improve service efficiency. Finally, it will truly realize the service optimization of the medical system by taking patients as the center.  Figure 1 is a conceptual diagram of smart medicine.

### 2.2. Subjects

100 hemodialysis patients admitted from May 2019 to May 2020 were randomly divided into the observation group and routine group with 50 cases each, and the MHD group with 50 cases, including 31 males and 19 females, aged from 15 to 56 years (36.1 5.9). The disease types were chronic renal failure (14 cases), chronic glomerulonephritis (20 cases), and chronic glomerulonephritis (20 cases). Conventional group includes 33 males and 17 females, aged from 15 to 56 years (37.0 6.0), and the types of diseases are 16 cases of chronic renal failure, 19 cases of chronic glomerulonephritis, and 15 cases of acute renal failure. The comparison of general data between the two groups is comparable, *P* > 0.05. The patient had no previous hypertension, hyperlipidemia, diabetes, heart disease, thyroid disease, blood disease, kidney disease, carotid artery intima-media thickening or plaque formation, no history of heavy drinking and smoking, and no abnormal biochemical indicators, electrocardiogram, chest X-ray, and echocardiography.

### 2.3. Methods

MHD group: ① Infection control management: strengthen the environmental management of departments, strictly limit the number of visitors in departments, regularly disinfect the items and environment in departments every day, keep indoor ventilation, strictly classify the medical wastes by nurses, carry out citric acid/sodium hypochlorite and heat elimination after dialysis, and destroy the used disposable materials in time. ② Infection prevention knowledge training: the head nurse regularly organizes department workers to participate in nursing knowledge training on hemodialysis infection control and regularly carries out technical assessment, so as to continuously improve nurses' subjective awareness of infection control and aseptic concept [10]. When entering the dialysis room during daily work, the medical staff are required to change sterile clothes and shoes, strictly abide by the dialysis treatment process, and standardize the nursing operation. Clinicians strictly limit the use of antibiotics. Routine group: routine health guidance, dialysis observation, medication guidance, and life care [11].

### 2.4. Instruments and Methods

The French Supersonic Imagine Aixplorer full digital color Doppler ultrasound diagnostic instrument with extreme speed imaging technology is adopted, and the probe frequency is 2–10 MHz. Instruct the examinee to take the supine position, fully expose the neck, and raise it behind the neck. First, routine carotid artery color Doppler ultrasound was performed to observe whether there were plaques in the lumen and the location and size of plaques. According to the ultrasound guidelines for blood vessels and superficial organs compiled by the Ultrasound Branch of Chinese Medical Doctor Association, plaques with thickness ≥1.5 mm and limitations protruding into the lumen are defined as plaques. According to the presence or absence of plaque, the MHD group was divided into the plaque group and plaque-free group. Make the probe parallel to the maximum section of the long axis of the common carotid artery, clearly display the carotid canal wall, and avoid the location of the carotid bulb and plaque. The rapid imaging technique was used to instruct the subjects to hold their breath, keep the subjects still, and keep the probe stable within 2 s. Then, measure the systolic start BS value and systolic end ES value of common carotid artery PWV [12].

### 2.5. Statistical Analysis

SPSS 20.0 statistical software was used to calculate, and the measurement data were expressed as mean ± standard deviation. The *t*-test was used for measurement data comparison, and the chi-square test was used for counting data comparison. *P* < 0.05 was considered statistically significant.

## 3. Results

Observation group consists of 31 males and 19 females, aged from 15 to 56 years (36.1 5.9), and the disease types are as follows: 14 cases of chronic renal failure, 20 cases of chronic glomerulonephritis, and 16 cases of acute renal failure. Routine group consists of 33 males and 17 females, aged from 15 to 56 years (37.0 6.0), and the disease types are as follows: 16 cases of chronic renal failure, 19 cases of chronic glomerulonephritis, and 15 cases of acute renal failure. The comparison of general data between the two groups is comparable.

### 3.1. Comparison of PWV between the Normal Control Group and MHD Group

The values of BS and ES in the normal control group were lower than those in the MHD group, and the difference was statistically significant (*P* < 0.05), as given in Table 1.

### 3.2. Comparison of PWV between the Plaque Group and Plaque Group in MHD Group

There was no significant difference in BS and ES values between the plaque group and the plaque group of MHD patients (*P* > 0.05), as given in Table 2.

### 3.3. Evaluate the Incidence of Infection in the Two Groups

The incidence of infection in the observation group was lower, *P* < 0.05, as given in Table 3.

### 3.4. Evaluate the Quality of Life of the Two Groups

**Figure 2:**
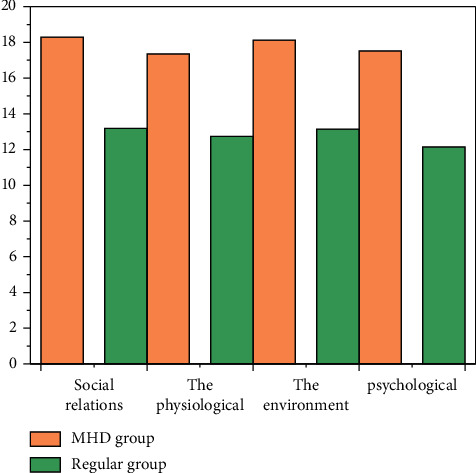
The quality of life scores between the two groups.

The incidence of catheter-associated infection in the intervention group was lower than that in the control group (*P* < 0.05). The incidence of exit infection and CRBSI in the intervention group was lower than that in the control group (*P* < 0.05), there was no significant difference in the incidence of tunnel infection between the two groups (*P* > 0.05), and the scores of quality of life in the observation group were all higher, *P* < 0.05, as shown in Figure 2.

## 4. Discussion

Cardiovascular complications are the most common complication in patients with chronic renal failure (CRF). Hyperlipoproteinemia, hypertension, hypoxia, and uremic toxin can all cause vascular endothelial function impairment in patients with CRF, thus reducing carotid artery elasticity. Increased vascular stiffness (decreased elasticity of large arteries) is a specific and sensitive marker of early vascular disease. The analysis found that the observation group adopted preventive nursing intervention, and the departments regularly organized personnel to participate in the training of infection prevention and control knowledge, which improved nurses' awareness of infection prevention, enhanced their initiative in nursing service, and actively sought for potential infection risks and actively prevented them during daily nursing work. In addition, preventive nursing service can constantly standardize nurses' nursing behavior, improving nurses' sterility awareness, and ensuring the safety and sterility of hemodialysis indoor environment through environmental maintenance, infection monitoring, and other measures, and ensuring that patients are in a safe state during hemodialysis can effectively reduce the risk of infection. In addition, strengthening diet management and health education can improve patients' immunity, promote patients' physical and mental health, and increase the times of nurse-patient communication. Maintaining good nurse-patient relationship can improve patients' risk awareness, improve patients' awareness of supervision of clinical nursing behavior, ensure the rationality of nursing behavior, actively reduce the risk of infection, and improve patients' quality of life [13, 14].

Traditional PWV mainly measures the propagation time of pulse wave between two baroreceptors, such as brachial-ankle, neck-femoral, neck-radial, femoral-ankle, and cardiac aorta and estimates the distance between them. However, for obese patients, it is difficult to operate, and the estimation of the distance will easily lead to errors, and it is difficult to measure the local vascular state, so it is difficult to obtain clinical recognition. With the emergence of ICT, Internet of Things, mobile Internet, data mining, and other information technologies, the combination of these advanced technologies with the medical and health field has improved the structure of medical resources and the quality of medical services for patients. Smart medicine is developing rapidly, and new conditions of medical and health have emerged [15]. The measured PWV is the BS value of the fastest time when the artery diameter increases and the ES value of the fastest time when the artery diameter decreases. The results showed that BS and ES values of PWV in MHD patients were significantly higher than those in the normal group, indicating that arterial elasticity decreased and arterial stiffness increased in MHD patients, which was consistent with previous reports. In addition, before there is anatomical evidence of atherosclerosis, abnormal vascular endothelial function can occur, which will cause the decrease of vascular elasticity and increase of vascular hardness, and can exist in the whole process of arteriosclerosis. In this study, the BS and ES values of MHD patients without plaque and patients with plaque were not significantly different, but were significantly higher than those of the normal group. It shows that the arterial stiffness of MHD patients has increased regardless of whether there is atherosclerotic plaque, and the functional change of arterial elasticity can be earlier than the structural change [16, 17].

In recent years, the medical Internet of Things is booming, and many hospitals are also using the medical Internet of Things technology, which is the core of smart medicine. RFID technology is especially widely used in the medical and health field, for example, RFID technology can be used for the management and application of the medical logistics system. RFID technology integrated electronic wristband, and medical records can be paperless application; RFID roll label integration patient bar code information can quickly identify the patient's identity. Some hospitals use mobile phones with THE function of PDA to exchange medical information, record necessary information at any time, write medical records, and give medical advice. Hospitals also use radiofrequency identification (RFID) technology to administer medications, dispense fluids, collect and process samples, and wear RFID tags to identify the baby's body size and prevent theft. Promote the development of intelligent medical treatment from the hospital perspective. Medical resources have not realized informationization and intelligence in the true sense. Electronic medical records, electronic prescriptions, and the electronization of hospital processes have not been widely implemented throughout the country. Telemedicine technology is not yet mature, and the number of intelligent medical equipment is limited. In view of these problems, hospitals should actively introduce advanced technology and talents, improve the level of telemedicine, and provide more advanced intelligent machinery and equipment. In addition, hospitals should actively construct an intelligent settlement mode of medical expenses, innovate service processes, simplify medical treatment links, and promote the application of mobile payment in the medical industry. Promote the development of smart medical treatment from the patient's point of view. Traditional medical treatment is deeply rooted in people's minds, and patients prefer to communicate with doctors face-to-face, which hinders the further development of the online medical platform to some extent. With the application of smart medicine, patients can enjoy one-stop medical service by using mobile medical app, which greatly facilitates people's lives. As patients, we should change our concepts, actively participate in it, and contribute to the development of wisdom medicine [18].

## 5. Conclusion

At present, there are mainly the following problems: the overall level of medical informatization construction is relatively backward, unreasonable allocation of medical resources, lack of technical talents for smart medical implementation, the medical and health system is not sound enough to meet the health needs of the people, medical big data cannot be fully utilized, and poor security of the medical network. Ultrasonography is safe, simple, noninvasive, nonradiation, fast, and so on. It can automatically and accurately detect carotid PWV and can quantitatively evaluate the degree of arteriosclerosis in MHD patients, which is beneficial to early intervention and treatment of cardiovascular dangerous events. It has a certain clinical application value for dynamic observation of disease changes and prognosis. Preventive nursing intervention can reduce the incidence of infection and improve the quality of life of hemodialysis patients.

## Figures and Tables

**Table 1:** Comparison of PWV between the control group and MHD group.

Group	BS	ES
MHD group	5.16 ± 0.99	5.84 ± 1.70
General group	7.18 ± 2.05	9.47 ± 3.87
*t* value	−2.366	−4.374
*P* value	0.019	0.000

**Table 2:** Comparison of PWV between the plaque group and plaque group in MHD patients.

Group	BS	ES
Group with plaque	5.66 ± 1.80	9.60 ± 2.87
No plaque group	6.40 ± 1.66	9.15 ± 2.95
*t* value	−1.415	0.550
*P* value	0.166	0.584

**Table 3:** Compare the infection between the two groups.

Group	Catheter infection	Skin infection	Respiratory tract infection	Incidence rate
MHD group	2	1	1	4
General group	4	3	3	10
*t*				5.980
*P*				0.014

## Data Availability

The data used to support the findings of this study are available from the corresponding author upon request.
